# Safety and Efficacy Performance of Coaxial 18G vs. 20G Needles for Pediatric Percutaneous Liver Biopsy: A Retrospective Cohort Study

**DOI:** 10.3390/jcm15093497

**Published:** 2026-05-02

**Authors:** Gil N. Bachar, Shlomit Tamir, Aeonv Choen, Yael Rapson, Ahuva Grubstein, Eli Atar

**Affiliations:** 1 Units of Vascular and Interventional Radiology, Department of Diagnostic Radiology, Rabin Medical Center, Hasharon and Beilinson Hospitals, Petach Tikva 49100, Israel; shlomittamir10@gmail.com (S.T.); neevush@gmail.com (A.C.); yael.rapson@gmail.com (Y.R.); ahuvag@clalit.org.il (A.G.); elia@clalit.org.il (E.A.); 2 Gray’s Faculty of Medicine, Tel Aviv University, Tel Aviv 6139001, Israel

**Keywords:** liver biopsy, coaxial needle gauge, ultrasound-guided biopsy, diagnostic yield, complications, pediatric interventional radiology

## Abstract

**Background:** Percutaneous liver biopsy is a cornerstone in the diagnostic and therapeutic management of pediatric liver diseases. However, data on the optimal needle gauge for coaxial techniques in children remain scarce. Smaller-gauge needles may theoretically enhance safety but could potentially compromise diagnostic yield. **Objectives:** The primary objective of this study was to evaluate and compare the safety and diagnostic clinical adequacy of ultrasound-guided percutaneous liver biopsies performed with semi-automated 20G versus 18G coaxial needles in pediatric patients. **Patients and Methods**: This retrospective cohort study included consecutive patients aged ≤19 years who underwent percutaneous non-targeted liver biopsies at a tertiary medical center between 2006 and 2012. Patient demographics, biopsy technique parameters (including needle gauge, number of cores, and tract embolization), and procedure-related complications were analyzed. Procedural success was defined by diagnostic and clinical adequacy, requiring a definitive pathology report and the presence of ≥7 portal tracts (the widely accepted threshold for a reliable histologic diagnosis). Complications were classified according to the Society of Interventional Radiology guidelines. **Results:** A total of 320 biopsies were performed in 260 patients (44.6% female; mean age 7.4 ± 6.0 years). Common indications included post-liver transplantation surveillance (28.4%) and unexplained liver enzyme elevation (22.5%). Biopsies were performed using 18G (n = 148; 46.3%) or 20G (n = 172; 53.7%) coaxial needles. Diagnostic and clinical adequacy was achieved in 100% of the procedures, with biopsy results directly influencing clinical management in 39.7% of cases. The overall complication rate was 5.3% (3.4% minor, 1.9% major), with no procedure-related mortality. While raw complication rates were numerically higher in the 20G group (likely to reflect an operator-driven selection bias for younger or higher-risk patients), the differences between the 18G and 20G needles were not statistically significant. Notably, the use of the 20G needle was associated with a significantly reduced clinical need for post-biopsy tract embolization. **Conclusions:** Our findings demonstrate no statistically significant differences in complication rates or diagnostic clinical adequacy between 18G and 20G coaxial needles for pediatric percutaneous liver biopsies. When selected based on appropriate clinical judgment, the 20G needle provides a high diagnostic yield and serves as an effective option, particularly for reducing the need for tract embolization. However, both 18G and 20G needles represent acceptable clinical options within the pediatric interventional armamentarium. Ultimately, the choice of needle gauge should be meticulously tailored to individual patient characteristics, bleeding risk profiles, and specific clinical indications, rather than uniformly recommending a smaller gauge across all pediatric age groups.

## 1. Introduction

Histopathological evaluation of the liver is crucial for the diagnosis, management, and appropriate investigation of a wide range of diffuse and focal liver diseases in pediatric patients. Percutaneous liver biopsy has become the standard technique for obtaining liver tissue for histological examination [1,2,3]. The success of this procedure is determined not only by the acquisition of sufficient tissue for histological diagnosis but also, and perhaps more importantly, by its safety profile.

Early studies of pediatric percutaneous liver biopsies indicated a higher rate of complications in very young children and immunocompromised patients, such as those post-transplant and oncology patients [2,4,5]. Furthermore, the use of ultrasound guidance has been demonstrated to enhance the safety profile of liver biopsies [1,6,7,8].

Based on the available pediatric literature, overall complication rates for percutaneous liver biopsy range from 1.9% to 11.8% [9,10,11,12,13,14], with major complications occurring in 1% to 2.4% of cases [3,15,16,17]. A large meta-analysis evaluating ultrasound-guided liver biopsies found that both 18-gauge and 20-gauge needles demonstrated exceptionally low major and minor complication rates [18]. However, the current literature suggests that factors other than needle gauge may be more significant predictors of complications in pediatric liver biopsies. These risk factors include an increased number of needle passes, young age (under 3 years) or low body weight (under 16 kg), underlying thrombocytopenia, and the use of native versus transplanted livers [16].

While direct comparative evidence regarding specific needle gauges in pediatric populations remains limited, one important study demonstrated that in children less than 5 years of age, the use of an 18-gauge spring-loaded needle was associated with a significantly higher change in hematocrit compared to aspiration needles [3]. This finding suggests that larger gauge needles may lead to more bleeding in younger children and that smaller gauge needles (such as 20G) might be preferable in this vulnerable age group.

The coaxial biopsy technique entails the insertion of a biopsy needle through a larger, outer guiding needle. While the coaxial approach offers potential advantages such as decreased capsule punctures and the option for tract embolization, pediatric data directly comparing the safety and efficacy of different coaxial needle gauges are currently lacking. Although previous pediatric literature highlights the safety of various non-coaxial needle sizes, there is a paucity of specific data regarding the optimal gauge for coaxial needles in this demographic [15,16,18,19,20,21,22,23,24,25].

The primary objective of this study was to evaluate and compare the safety and diagnostic clinical adequacy of 20G vs. 18G coaxial needles for percutaneous liver biopsies in pediatric patients. Previous literature suggests that larger-gauge biopsy needles may be associated with a higher incidence of bleeding complications [16,20,26,27,28]. We posited that employing a smaller-gauge coaxial needle (20G) for pediatric liver biopsy would enhance safety without compromising diagnostic accuracy when compared to 18G coaxial needles.

## 2. Patients and Methods

### 2.1. Study Population

The cohort comprised all consecutive patients who met the following inclusion criteria: age ≤ 19 years and having undergone percutaneous liver biopsy at a tertiary university-affiliated medical center between 1 June 2006 and 30 April 2012. Exclusion criteria included biopsies of focal liver lesions, non-percutaneous procedures, and cases with insufficient medical documentation.

### 2.2. Procedure

As previously described, experienced interventional radiologists conducted all biopsies under ultrasound guidance using the Zonare One ultrasound system (Zonare Medical Systems, Mountain View, CA, USA). The choice of transducer was tailored to the patient’s size: a 2–6 MHz convex probe was used for most cases, while a 5–14 MHz linear probe was utilized for infants to achieve higher spatial resolution [1,6]. Informed consent for the biopsy procedure was obtained from the parents or legal guardians of all participants. For this retrospective study of medical records, the Institutional Review Board granted a waiver of informed consent. Coagulation parameters (platelet count and INR) were corrected when necessary. Coagulation parameters were corrected to achieve a target International Normalized Ratio (INR) of ≤1.5 and a platelet count of ≥50,000/µL before the procedure, in accordance with our institutional protocol. All biopsies were performed under general anesthesia, which was administered and continuously monitored by a dedicated pediatric anesthesiologist. The anesthesia protocol was tailored to the patient’s age and level of cooperation. In older, cooperative children, the procedure was typically performed under intravenous propofol alone, supplemented with local anesthetic infiltration (lidocaine) along the planned needle tract. Conversely, for infants and younger children, general anesthesia was induced and maintained via inhaled sevoflurane. In this younger cohort, airway management was primarily secured using a laryngeal mask airway (LMA), and local anesthesia with lidocaine was similarly administered. When specifically indicated by the patient’s clinical condition, endotracheal intubation and short-acting intravenous analgesics (e.g., fentanyl) were utilized.

A subxiphoid anterior approach (segment 4) was preferred, although the exact entry site was at the operator’s discretion. Local anesthesia with 1% lidocaine and a ~2 mm skin incision was utilized. Biopsies were performed using semi-automated coaxial core-needle systems: outer needles 17G or 19G, with corresponding inner biopsy needles of 18G or 20G, from various manufacturers. When blood was observed within the outer coaxial needle, or when INR ≥ 1.5 or platelets ≤ 100,000/µL, the needle tract was prophylactically embolized using Gelfoam particles to provide an additional safety measure for these high-risk patients with underlying hemostatic dysfunction, despite pre-procedural correction, as described by Amaral [4]. Outpatients were routinely monitored for 24 h post-procedure.

### 2.3. Data Collection

The study was conducted in accordance with the Helsinki Declaration and approved by the local Institutional Review Board. Retrospective data were extracted from the patients’ angiography logs, electronic medical records, and gastroenterology clinic archives. The variables collected included demographics (age, date of biopsy), technical details (needle gauge, number of cores, and tract embolization), pathology (diagnostic yield and number of portal tracts), and clinical outcomes (indication, complications, and impact on management). Procedural success was defined based on diagnostic and clinical adequacy rather than strictly standardized histological parameters. A biopsy was considered diagnostically and clinically adequate if the obtained specimen yielded a definitive histopathological diagnosis that directly informed or guided clinical management without necessitating a repeat biopsy due to insufficient tissue yield [22,23]. Following the Society of Interventional Radiology (SIR) guidelines, complications were stratified into major and minor categories [18]. Major complications included those necessitating blood transfusion, ICU admission, surgery, prolonged hospitalization (≥48 h), permanent damage, or death.

### 2.4. Statistical Analysis

Descriptive statistics were used for the analysis. Continuous variables are presented as mean ± standard deviation (SD) or median with interquartile range (IQR). Categorical variables were expressed as frequencies and percentages. Chi-square tests were employed to compare categorical data, and chi-square tests were used to calculate the *p*-value between major and minor complications. Complication and success rates were reported with 95% confidence intervals. Categorical variables were compared using the chi-square test or Fisher’s exact test when expected cell counts were less than 5. Sample size calculation was not conducted, as all eligible biopsies over six years were included in the study. A 95% confidence interval was used. Statistical significance was set at *p* < 0.05. All statistical analyses were performed using SPSS version 25.0 (SPSS Inc., Chicago, IL, USA).

## 3. Results

### 3.1. Cohort Description

A total of 320 percutaneous liver biopsies were performed in 260 pediatric patients between January 2006 and April 2012. Table 1 presents the distribution of the number of biopsies per patient. Most patients (85.8%, n = 223) underwent a single biopsy, while the remainder underwent 2–6 biopsies. Notably, three patients who underwent five or more biopsies were liver transplant recipients. Figure S1 illustrates the detailed patient selection process, outlining the exclusion criteria and the final study cohort of 260 patients who underwent a total of 320 percutaneous liver biopsies, stratified by needle gauge.

The cohort comprised 116 females (44.6%) and 144 males (55.4%). The age of participants ranged from 18 days to 18 years and 11 months, with a mean age of 7.4 ± 6 years and a median age of 5.9 years (IQR: 1.9–12.6 years). The mean age at the time of the first biopsy was 7.3 ± 6.1 years. No discernible temporal trends were observed in the annual number of biopsies. When evaluating the baseline characteristics of the cohort stratified by needle size, an expected operator-driven selection bias was observed. Specifically, patients who underwent biopsy with the smaller 20G needle were generally younger and of smaller body habit compared to those in the 18G group. This difference reflects the standard clinical practice of preferentially selecting a smaller gauge for infants, younger children, and higher-risk patients in an effort to minimize potential procedural complications.

### 3.2. Indications

Table 2 details the indications for biopsies, encompassing a total of 320 cases. The most prevalent indication for biopsy was post-liver transplantation (n = 92, 28.7%), followed by unexplained elevation of liver enzymes (n = 72, 22.5%). Additional indications included inflammatory bowel disease, chronic kidney disease, diabetes, immunodeficiency, hematologic/oncologic disorders, and pre- or post-transplantation monitoring.

All biopsies were performed coaxially using inner needles of either 18G or 20G: 148 (46.3%) with 18G and 172 (53.7%) with 20G.

### 3.3. Core Numbers and Tract Embolization

The number of cores obtained ranged from 2 to 7, with a median value of 3. Gelfoam tract embolization was employed in 51.9% of cases, predominantly in 18G biopsies (90% compared to 20% for 20G biopsies).

### 3.4. Diagnostic Yield

All biopsies satisfied the criteria for technical adequacy, achieving a 100% success rate in each case. Four repeat biopsies were conducted: two due to suspected sampling error despite adequate material, one owing to early non-diagnostic sampling in an infant with biliary atresia, and one with unclear documentation. No statistically significant differences in repeat rates were observed between the 18G and 20G groups.

### 3.5. Clinical Impact

Biopsy results influenced clinical management in 39.7% of cases, most frequently in the context of autoimmune hepatitis, infectious workup, neonatal cholestasis, and post-transplant assessment. Table 3 describes the impact of pathologic biopsy results on patient management by indication.

Statistical analysis using the chi-square test demonstrated a highly significant association between the clinical indication and the biopsy’s effect on management (*p* < 0.001). High rates of therapeutic modification were observed in autoimmune hepatitis (64.7%), infectious workup (60.9%), and neonatal cholestasis (55.2%). In contrast, indications such as fulminant hepatic failure (29.4%) and liver disorder NOS (26.4%) were more likely to result in no change to the pre-existing clinical plan.

### 3.6. Complications

The overall complication rate was 5.3% (95% CI: 2.85–7.75%), comprising 3.4% minor and 1.9% major complications. No procedure-related mortality was reported. One patient required surgical intervention due to a biliary leak following concomitant cholecystostomy, and three patients required blood transfusion, with only one directly attributable to biopsy-related hemorrhage. Comparing the two needle gauges, the 20G needle shows a slightly higher percentage of both minor (4.0% vs. 2.7%) and major (2.9% vs. 0.7%) complications compared to the 18G needle, based on the provided raw counts and percentages. However, as previously analyzed using the chi-square test, these differences were not statistically significant for either major or minor complications at a 0.05 significance level. The complication rates did not differ significantly between the 18G and 20G groups (*p* = 0.29 and 0.71, respectively) (Table 4). No significant associations were identified between complications and variables such as age, indication, core number, or tracheostomy.

## 4. Discussion

Our study did not reveal any statistically significant differences in safety or efficacy between the use of 20G and 18G coaxial needles for percutaneous liver biopsies in pediatric patients. Technical success was achieved in all biopsy procedures. In four instances where a repeat biopsy was necessary, two were attributed to suspected sampling errors rather than insufficient material. In the remaining two cases, the medical records did not specify whether the non-diagnostic result of the initial biopsy was due to technical issues (such as insufficient tissue) or other factors (e.g., early biopsy with limited pathological findings).

The overall complication rate was 5.3%, with 1.9% of procedures resulting in major complications. Comparing the two needle gauges, the raw complication rates were numerically higher in the 20G group for both minor (4.0% vs. 2.7%) and major (2.9% vs. 0.7%) complications. This numerical difference likely reflects the inherent selection bias of our study, wherein operators preferentially reserved the smaller 20G needles for more vulnerable or higher-risk patients. Our analysis revealed no statistically significant differences in complication rates between the 20G and 18G needles, nor was any association identified between complications and other parameters, such as age, biopsy indication, or the number of cores obtained. However, importantly, given the retrospective and non-randomized nature of this study, this lack of statistical significance should not be interpreted as definitive evidence of clinical equivalence, nor does it imply the superiority of the smaller needle.

These rates are comparable to those reported in the literature for larger diameter and non-coaxial needles. In Hatfield’s study [9], the complication rate for both coaxial and non-coaxial needles (18G and 20G) was 3.2%, with 0.8% classified as major complications. No statistically significant difference was found between the coaxial and non-coaxial techniques. In that study, 5% of all liver and kidney biopsies were performed using 20G needles; however, the complication rates for 20G liver biopsies were not reported.

Scheimann et al. [3] examined liver biopsies using non-coaxial needles ranging from 15G to 18G, reporting an overall complication rate of 6.83%, including 2.4% major complications. No statistically significant differences were observed between the different needle types. However, they noted an increased incidence of bleeding complications in children under five years of age when using ASAP spring-loaded needles (non-coaxial). The small cohort size and potential selection bias may have influenced their results. Additionally, some biopsies in their cohort were performed on specific lesions, in contrast to our study, which exclusively included non-targeted liver biopsies. As indicated, we did not identify any statistically significant association between patient age and complication rates.

Two studies by Nabili et al. [1,6] assessed the safety and efficacy of ultrasound-guided biopsies and concluded that sonographic guidance significantly enhanced biopsy performance. Their adequacy criteria included a core length of ≥15 mm and ≥10 portal tracts in the specimen. In 421 biopsies conducted using non-coaxial 18G needles with a single pass in 80% of cases, they reported a 100% technical success rate and no bleeding or other major complications; however, the precise definition of a complication was not specified. Within our cohort of 320 biopsies, only one case of severe bleeding was directly attributable to the biopsy. Sornsakrin et al. [10] investigated biopsies in a subgroup of pediatric liver transplant recipients, reporting an overall complication rate of 5%, including 1.7% major complications. The needle gauge was not specified in their reports. This complication rate is comparable to ours, possibly because liver transplantation was the most common indication in our study. However, no statistically significant association was identified between complication rates and indications such as liver transplantation or bone marrow transplantation (both higher-risk populations).

Hoffer et al. [2] concentrated on a subgroup of pediatric oncology patients, including those who had undergone bone marrow transplantation. Of the 22 percutaneous liver biopsies, 21 were coaxial (21 with 17G and one with 14G). Tract embolization was performed in all patients. They reported a 100% technical success rate, with three of 22 cases experiencing bleeding complications, including one major complication that necessitated the transfusion of blood products. Amaral et al. [4] examined the complication rates of biopsies performed on children under one year of age. The majority of these biopsies utilized non-coaxial 18G needles, with only seven out of 65 biopsies employing coaxial 17G needles. Tract embolization was performed in all cases. The overall complication rate was 9.2%, with major complications occurring in 4.6% of cases, suggesting higher complication rates in infants. In our study, 49 infants under one year of age were included; 16 underwent biopsy with a 20G coaxial needle (needle gauge data were unavailable for 20 cases). The overall complication rate in this cohort was 8.2% (n = 4), with two (4.1%) major complications. In summary, a review of the literature indicates that the overall complication rate for pediatric percutaneous liver biopsies ranges from 0% to 9.2%. The results of our study are consistent with the reported range.

In our study, liver biopsy results directly influenced clinical management in 39.7% of cases, a rate comparable to other pediatric series, while the remaining 60.3% of procedures provided substantial clinical value through diagnostic reassurance and confirmation of existing treatment strategies. A significant portion of these procedures involved post-transplant protocol biopsies or surveillance, where histological findings of a stable allograft or resolved rejection provided critical validation of the current immunosuppressive regimen. Furthermore, for patients with unexplained liver enzyme elevation (22.5%), ‘negative’ or non-specific histological findings were crucial for ruling out aggressive pathologies, such as autoimmune hepatitis or metabolic disorders, thereby justifying a conservative observational approach. Thus, the clinical value of pediatric liver biopsy in our cohort encompassed both active therapeutic modification and the essential role of diagnostic confirmation and risk stratification. The clinical impact was etiology-dependent, with the highest management modification rates observed in patients with suspected AIH, infectious workup, and neonatal cholestasis, whereas in the post-transplant population, the biopsy primarily served as a critical tool for rejection grading and treatment validation.

A noteworthy finding in our study was the significant difference in the utilization of Gelfoam tract embolization between the two needle sizes. Gelfoam was employed in 90% of the 18G biopsies, compared to only 20% in the 20G group. This discrepancy suggests that the 20G needle allowed for a less invasive procedure with a reduced perceived need for additional hemostatic interventions. In pediatric interventional radiology, where the objective is to minimize procedural complexity and tissue trauma, the ability to achieve a 100% technical success rate and comparable diagnostic yield with a smaller needle and without routine tract embolization is a significant clinical advantage. Therefore, although statistical analysis did not show a difference in major complication rates, the 20G coaxial needle aligns more closely with the principle of minimal invasiveness (ALARA) while maintaining high diagnostic efficacy across all pediatric age groups. Moreover, pediatric patients, particularly neonates and infants (who comprised a significant portion of our cohort, with a median age of 5.9 years), possess smaller liver volumes and face higher procedural risks associated with large-bore needles. While the raw complication rates were slightly higher in the 20G group, the differences were statistically insignificant (*p* = 0.29 and *p* = 0.71). Consequently, if a thinner needle does not incur a clinical penalty regarding diagnostic yield, it should logically be considered a primary choice to reduce the procedural footprint.

In our study, a biopsy was considered technically adequate if a diagnostic pathology report was obtained and if there were ≥7 portal tracts present. However, the American Association for the Study of Liver Diseases (AASLD) and the Society of Interventional Radiology advocate for a more stringent criterion of ≥11 complete portal tracts and a specimen length of 20–25 mm for optimal grading and staging of chronic liver disease [19,23,24,25]. Nevertheless, these targets are often difficult to achieve in the pediatric population due to safety considerations and smaller liver volumes. In clinical practice, a minimum of 6–7 portal tracts is widely accepted as sufficient for a reliable histologic diagnosis. Consequently, our criteria for repeat biopsy were based on whether the initial specimen was insufficient, particularly in cases where the sample contained fewer than 6–7 portal tracts or was too fragmented to permit a definitive diagnosis, grading, or staging. This approach ensures that clinical management is guided by samples providing diagnostic clarity while also respecting the technical and safety constraints inherent in pediatric percutaneous procedures [19].

Our study has several limitations, primarily stemming from its retrospective design. First, the retrospective nature resulted in some missing data, the inclusion of which might have slightly altered the findings.

Second, our statistical analysis was conducted on a per-procedure basis (320 biopsies) rather than a per-patient basis (260 patients). While complications are largely procedure-specific, treating repeated biopsies in the same patient as completely independent observations violates the strict statistical assumption of independence. This clustering effect could potentially underestimate the variability in complication rates, particularly if certain patients had an inherent, persistent predisposition to bleeding or other adverse events.

Third, there is an inherent selection bias due to the non-randomized design. The choice between an 18G and a 20G needle was made at the operator’s discretion, heavily influenced by patient-specific factors such as age, body habits, and coagulation profile. Therefore, our findings should be interpreted as a validation of appropriate, individualized patient triage rather than a direct randomized comparison of needle efficacy. Nevertheless, the data firmly support the 20G coaxial system as a robust tool in the pediatric interventional armamentarium.

Fourth, procedural success was defined based on diagnostic and clinical adequacy rather than strict, standardized histological criteria (e.g., a specific specimen length or a minimum number of portal tracts), as these precise quantitative metrics were not uniformly available in the historical pathology reports. We acknowledge that relying on this clinical composite definition may potentially yield higher reported success rates than evaluating purely histological adequacy, as some specimens may be diagnostically sufficient for specific clinical indications despite suboptimal histological dimensions.

Fifth, the retrospective data collection constrains our ability to precisely evaluate minor complications and the exact timing of complication onset following a biopsy. This factor influences recommendations for post-biopsy monitoring; while this issue has been previously examined by guidelines from the American Gastroenterological Association [5] and the Society of Interventional Radiology [26], our institutional surveillance protocols were more stringent.

Sixth, a significant limitation inherent to our retrospective design is the presence of uncontrolled confounding variables. Specifically, variables such as exact patient age, underlying comorbidities, disease severity, and differences in individual operator experience were not fully controlled for in our analysis. Furthermore, due to the very low absolute number of adverse events (particularly major complications), we were unable to perform a robust multivariable logistic regression to adjust for these potential confounders, as such a model would be statistically underpowered and prone to overfitting.

Finally, our cohort exclusively included non-targeted liver biopsies, excluding patients who underwent targeted biopsies for focal hepatic lesions, which may present a different procedural risk profile.

This study offers several significant advantages that contribute to the clinical understanding of pediatric liver biopsies. First, it encompasses a large and diverse cohort of 320 biopsies over seven years, providing robust real-world data on both 18G and 20G coaxial needles. Second, by demonstrating that the 20G needle maintains a 100% technical success rate and comparable diagnostic yield to the 18G needle, our findings support the adoption of a more minimally invasive approach without compromising pathological accuracy. Another key advantage is the evidence showing that the 20G needle significantly reduces the clinical need for Gelfoam tract embolization, thereby simplifying the procedure. Furthermore, the inclusion of a wide range of indications, including a substantial transplant population, enhances the generalizability of our results to various pediatric hepatological practices. Ultimately, this study underscores the reliability of standardized, ultrasound-guided coaxial techniques across multiple operators, reinforcing the safety and efficacy of smaller gauges.

In conclusion, our retrospective cohort study demonstrated no statistically significant differences in complication rates or diagnostic and clinical adequacy between the 18G and 20G coaxial needles for pediatric percutaneous liver biopsies. When selected based on appropriate clinical judgment, the 20G needle provides a high diagnostic yield and serves as an effective option, particularly for reducing the need for post-biopsy tract embolization. However, rather than uniformly recommending the smaller needle gauge across all pediatric age groups, the lack of statistically significant differences in complication rates indicates that both 18G and 20G needles represent acceptable clinical options. Ultimately, the choice of needle gauge should continue to be meticulously tailored to individual patient characteristics, bleeding risk profiles, and specific clinical indications. The outcomes of this study may aid our Interventional Radiology Unit in formulating definitive guidelines for liver biopsies in pediatric and potentially adult populations.

## Figures and Tables

**Table 1:** Number of biopsies per patient.

No of Biopsies	Frequency	Percent
1	223	85.8
2	23	8.8
3	9	3.5
4	2	0.8
5	2	0.8
6	1	0.4
Total	260	100.0

**Table 2:** Biopsy Indications.

Biopsy Indications		
	Frequency	Percent
Post Liver Transplant	92	28.7
Liver Disorder NOS	72	22.4
Neonatal Cholestasis	29	9.0
Infectious (HCV, HBV, CMV)	23	7.2
Post BMT	21	6.5
Fulminant Hepatic Failure	17	5.3
Autoimmune Hepatitis (suspected or proven in previous biopsy)	17	5.3
Portal Hypertension	4	1.2
Metabolic Disease	3	0.9
Deferral Treatment for Thalassemia	3	0.9
Familial Mediterranean Fever (FMF)	3	0.9
Fever of Unknown Origin (FUO)	2	0.6
Total Parenteral Nutrition (TPN)	2	0.6
Other	33	10.3
Total	321	100.0

Other: includes children with elevated liver enzymes and other underlying diseases such as inflammatory bowel disease, chronic kidney failure, diabetes, immunodeficiency, and hemato-oncologic diseases. NOS—not otherwise specified. HCV—hepatitis C virus. HBV—hepatitis B virus. CMV—cytomegalovirus. BMT—bone marrow transplantation.

**Table 3:** The effects of pathologic results on patient management per indication.

Indication Total	Effect			*p*-Value
	Yes	No	Unknown	
Autoimmune Hepatitis 17 (suspected or proven in previous biopsy)	11 (64.7%)	4 (23.5%)	2 (11.8%)	<0.001
Infectious (HCV, HBV, CMV) 23	14 (60.9%)	8 (34.8%)	1 (4.3%)	<0.001
Neonatal Cholestasis 29	16 (55.2%)	12 (41.4%)	1 (3.4%)	<0.001
Post Liver Transplant 91	47 (51.6%)	43 (47.3%)	1 (1.1%)	
Fulminant Hepatic Failure 17	5 (29.4%)	12 (70.6%)	0	
Liver Disorder NOS 72	19 (26.4%)	48 (66.7%)	5 (6.9%)	
Post BMT 21	2 (9.5%)	1 (4.8%)	18 (85.7%)	
Fever of Unknown Origin (FUO) 2	1 (50%)	1 (50%)	0	
Portal Hypertension 4	0	3 (75%)	1 (25%)	
Familial Mediterranean Fever (FMF) 3	0	3 (100%)	0	
Metabolic Disease 3	1 (33.3%)	1 (33.3%)	1 (33.3%)	
Deferoxamine Treatment for Thalassemia 3	0	0	3 (100%)	
Total Parenteral Nutrition (TPN) 2	2 (100%)	0	0	
Other 33	9 (27.3%)	21 (63.6%)	3 (9.1%)	
Total 320	127 (39.7%)	157 (49.1%)	36 (11.2%)	

Other: includes children with elevated liver enzymes and other underlying diseases such as inflammatory bowel disease, chronic kidney failure, diabetes, immunodeficiency, and hemato-oncologic diseases. NOS—not otherwise specified. HCV—hepatitis C virus. HBV—hepatitis B virus. CMV—cytomegalovirus. BMT—bone marrow transplantation.

**Table 4:** Complication rate by needle gauge.

	Complications Total			
	None	Minor	Major	
18G	143 (96.6%)	4 (2.7%)	1 (0.7%)	148
20G	160 (93%)	7 (4.0%)	5 (2.9%)	172
Total	302 (94.4%)	11 (3.4%)	6 (1.9%)	320

The *p*-value for the difference in general (minor and major) complication rate between 18G and 20G is 0.29 and 0.71 (non-significant). The 95% confidence interval for the general complication rate in the total sample is 2.85–7.75%.

## Data Availability

The datasets used and/or analyzed during the current study are available from the corresponding author upon reasonable request.

## Supplementary Materials

The following supporting information can be downloaded at: https://www.mdpi.com/article/10.3390/jcm15093497/s1, Figure S1: Flowchart of the patient selection process and procedure stratification.
