# Supplementary figures and images for: Safety and Efficacy Performance of Coaxial 18G vs. 20G Needles for Pediatric Percutaneous Liver Biopsy: A Retrospective Cohort Study
Source: J Clin Med. 2026 May 2;15(9):3497. doi: 10.3390/jcm15093497 (PMC13164113; doi:10.3390/jcm15093497)

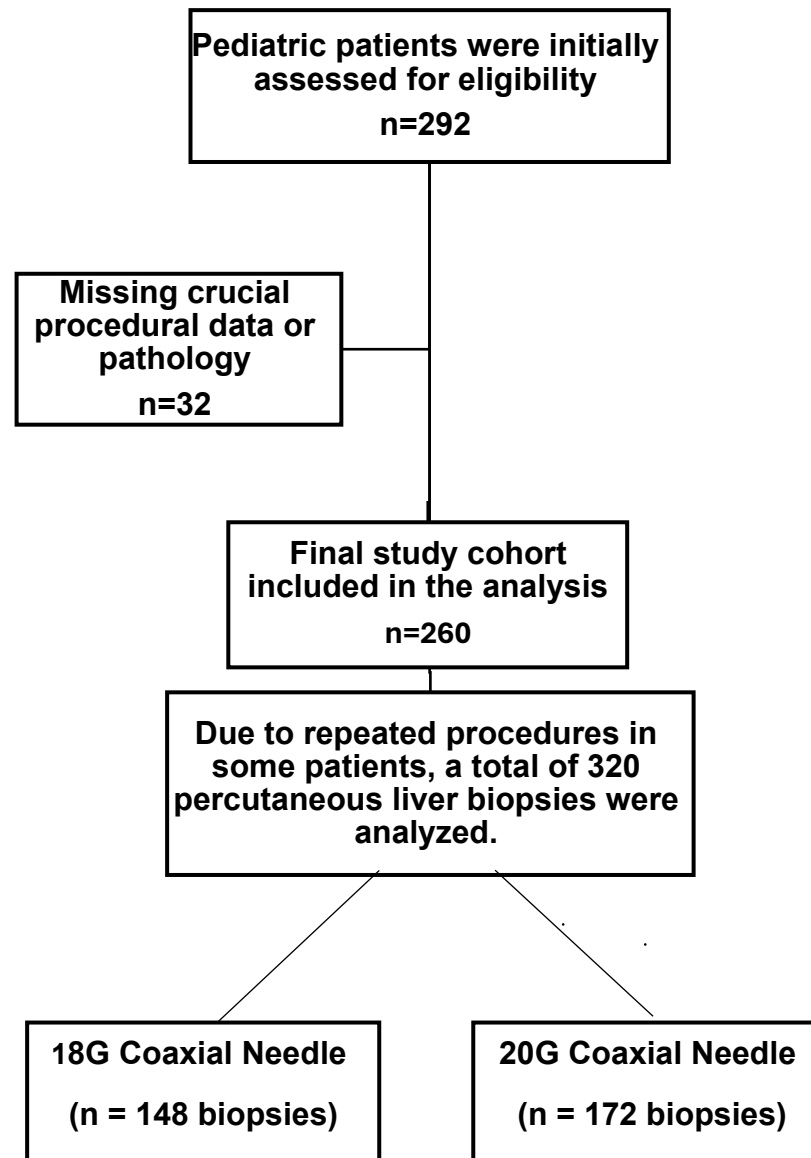

Figure S1: Flowchart of the patient selection process and procedure stratification

Supplement: Supplementary file 1 [file jcm-15-03497-s001.zip › jcm-4168660-supplementary.pdf]
